# Genetic factors associated with patient-specific warfarin dose in ethnic Indonesians

**DOI:** 10.1186/1471-2350-12-80

**Published:** 2011-06-06

**Authors:** Ivet M Suriapranata, Wen Ye Tjong, Tingliang Wang, Andi Utama, Sunu B Raharjo, Yoga Yuniadi, Susan SW Tai

**Affiliations:** 1 Mochtar Riady Institute for Nanotechnology, Tangerang, Indonesia; 2 Harapan Kita Hospital, National Cardiovascular Center, Department of Cardiology, Faculty of Medicine, University of Indonesia, Jakarta, Indonesia

**Keywords:** Warfarin, SNP, *CYP2C9*, *VKORC1*, Indonesia

## Abstract

**Background:**

*CYP2C9 *and *VKORC1 *are two major genetic factors associated with inter-individual variability in warfarin dose. Additionally, genes in the warfarin metabolism pathway have also been associated with dose variance. We analyzed Single Nucleotide Polymorphisms (SNPs) in these genes to identify genetic factors that might confer warfarin sensitivity in Indonesian patients.

**Methods:**

Direct sequencing method was used to identify SNPs in *CYP2C9, VKORC1, CYP4F2, EPHX1, PROC *and *GGCX *genes in warfarin-treated patients. Multiple linear regressions were performed to model the relationship warfarin daily dose requirement with genetic and non-genetic variables measured and used to develop a novel algorithm for warfarin dosing.

**Results:**

From the 40 SNPs analyzed, *CYP2C9 *rs17847036 and *VKORC1 *rs9923231 showed significant association with warfarin sensitivity. In our study population, no significant correlation could be detected between CYP2C9*3, CYP2C9C-65 (rs9332127), *CYP4F2 *rs2108622, *GGCX *rs12714145, *EPHX1 *rs4653436 and *PROC *rs1799809 with warfarin sensitivity.

**Conclusions:**

*VKORC1 *rs9923231 AA and *CYP2C9 *rs17847036 GG genotypes were associated with low dosage requirements of most patients (2.05 ± 0.77 mg/day and 2.09 ± 0.70 mg/day, respectively). *CYP2C9 *and *VKORC1 *genetic variants as well as non-genetic factors such as age, body weight and body height account for 15.4% of variance in warfarin dose among our study population. Additional analysis of this combination could allow for personalized warfarin treatment in ethnic Indonesians.

## Background

Warfarin is the most widely used oral anticoagulant in the world. It is usually prescribed for treatment of atrial fibrillation, heart valve prosthesis, recurrent stroke, deep vein thrombosis and pulmonary embolism [1]. Although warfarin is indispensable for treatment of thromboembolism and for prophylaxis of stroke, due to the large inter-individual variation in the requirement for this drug the appropriate dose to each patient is not easily adjustable. An insufficient dose will result in failure to prevent thrombosis, while overdose increases the risk of unexpected bleeding. Pharmacogenetic differences are believed to cause the variation in individual response to warfarin [1,2].

Warfarin is primarily metabolized to the 7-hydroxylated form in humans, principally by cytochrome P450 *2C9*. So far, more than 30 variant alleles in the *CYP2C9 *gene have been described (http://www.cypalleles.ki.se/cyp2c9.htm). Two common allelic variants CYP2C9*2 (rs1799853) and CYP2C9*3 (rs1057910) are among the most well characterized of the *CYP2C9 *alleles; both alleles have been associated with reduced enzymatic activity, and thus with reduced warfarin metabolism. Individuals bearing variant alleles CYP2C9*2 and *3 are reported to require a lower maintenance dose of warfarin and a longer time to achieve stable dosing. These individuals are also reported to have a higher proportion of prothrombin-time measurements above therapeutic range, and to experience more frequent bleeding events as compared to individuals with the CYP2C9*1 wild-type allele [3].

Variability in warfarin response is also attributable to variants in the vitamin K-related gene. Warfarin makes use of its anticoagulant effects by interfering with regeneration of vitamin K by reduction of its 2,3-epoxide in the vitamin K cycle, leading to inhibition of gamma-carboxylation of vitamin K-dependent clotting factor II (prothrombin), VII, IX and X. Several reports have demonstrated that non-coding SNPs in the vitamin K related gene influenced warfarin sensitivity. These data strongly suggest that differences in genetic variations in both *CYP *and vitamin K-related genes could explain the diversity in warfarin sensitivity and dose requirement [2-4].

Many reports indicated an additive effect on warfarin dose requirement in combinations of variant forms of *VKORC1 *and *CYP2C9 *genes in Caucasian patients. Furthermore, it is well known that there are significant differences in warfarin dose requirement in different ethnic groups; for example, Chinese patients were reported to require a warfarin dose nearly 40% lower than that required by Caucasian patients. The therapeutic maintenance dose of warfarin for Japanese was also 31% lower than that for Caucasians [5].

Recent genome wide association studies have confirmed known polymorphisms in *CYP2C9 *and *VKORC1 *as the primary genetic determinants of stabilized warfarin dose. Variants in both genes have been reported to cause ~40% of the variability in warfarin dose. In addition SNP rs2108622 of *CYP4F2 *was reported to contribute to 1%-2% of the variability [6,7].

In this study we investigate genetic variants previously identified to associate with warfarin metabolism in other populations, in order to identify genetic variations that might confer sensitivity to warfarin in Indonesian patients. Based on the gene variants, which are significantly associated with warfarin sensitivity, we aim to develop a prediction system that will be able to determine the dose appropriate to each patient in Indonesia.

## Methods

### Study population

All patients were unrelated ethnic Indonesians treated at the National Cardiovascular Centre, Harapan Kita Hospital, Jakarta, Indonesia for thromboembolic diseases, such as atrial fibrillation, valve replacement, atherothrombotic diseases or congestive heart failure from October 2007 to November 2008. All patients had a stable dosage of warfarin for minimum 3 months and an International Normalized Ratio (INR) of the Thrombo Test within the range of 1.5-3.0. Patients with incomplete history of warfarin medication were excluded in this study. Information about gender, age (missing in two patients), bodyweight, body height (missing in two patients), treatment indication and other diseases was taken from the patients' medical records. The ethical committee of Harapan Kita Hospital, Jakarta, approved the study. Prior to their participation in this study all patients have given their written informed consents. For genotyping purposes 122 subjects were included in this study; of which 85 samples with complete medical information were used in the statistical analysis for association with warfarin dose requirement.

### DNA isolation and genotyping

Genomic DNA was extracted from 200 μL whole blood using Illustra Blood GenomicPrep Mini Spin Kit (GE Healthcare, Buckinghamshire, UK) and QIAamp DNA Blood Mini Kit (Qiagen, Hilden, Germany) according to manufacturer's standard protocol. The genomic DNA concentration was measured using NanoDrop™ Spectrophotometer (Thermo Fisher Scientific, USA) and adjusted to 10 ng/μl.

The initial screening of SNPs was performed using 122 samples for partial *CYP2C9 *locus, which included Promoter regions, Exon 1, Exon 2, Exon 3, Exon 4, Exon 5, Exon 6, Exon 7, Exon 8 and Exon 9. Genotyping was also performed for *VKORC1 *(rs9923231); *EPHX1 *(rs4653436); *GGCX *(rs12714145); *PROC *(rs2069920; rs1799808; rs1799809); and *CYP4F2 *(rs2108622). Primers used for *CYP2C9 *and *VKORC1 *genotyping were designed according to Ref. [7,8]. The nucleotide position for the primers were designed according to corresponding published sequence in the NCBI database using Primer3 software (GenBank accession number NG_008385 for *CYP2C9*, NG_011564 for *VKORC1*, NG_011811.1 for *GGCX*, NG_009776 for *EPHX1*, M11228 for *PROC *and NG_007971 for *CYP4F2; *Primer3 is available at http://frodo.wi.mit.edu/primer3/).

The primer sequences were listed in Additional file 1: Table S1. Approximately 30 ng of total DNA were subjected to Polymerase Chain Reaction (PCR) amplification in a final volume of 25 μL. Each PCR reaction contained 1× PCR buffer (AmpliTaq^® ^Gold, Applied Biosystems, Foster City, CA), 1.5 mM MgCl_2_, 200 μM dNTPs, 0.4 μM forward and reverse primers, and 0.625 U HotStart *Taq *Polymerase (Applied Biosystems, Foster City, CA). The PCR were carried out in a Bio-Rad PTC-200 DNA Engine Cycler with an initial denaturation step of 10 min at 95°C, followed by 45 cycles of denaturation at 94°C for 30 s, annealing for 30 s (annealing temperature of each primer were listed in Additional file 1: Table S1), and extension at 72°C for 1 min, with a final extension for 10 min at 72°C. The purity and mobility of each PCR product were confirmed in 2% agarose gel electrophoresis; each PCR product was either purified using Multi Screen PCR_96 _Filter Plate (Millipore, Billerica, MA) or subjected to incubation with 1 U of Shrimp Alkaline Phosphatase (New England Biolabs, Ipswich, MA) and 1 U of Exonuclease I (New England Biolabs, Ipswich, MA). Subsequently each PCR product was sequenced using BigDye Terminator v3.1 Cycle Sequencing Kit and ABI Prism^® ^3130xl Genetic Analyzer (Applied Biosystems, Foster City, CA) according to manufacturer's standard protocol. Each sample was sequenced for both strands. All sequencing reactions were performed at least twice with DNA amplified from at least two independent PCR. Sequencing results were aligned and analyzed for SNPs using BioEdit 7.0.0 program (Applied Biosystems, USA). All SNPs detected in this study were located within the high quality region of the chromatogram.

### Statistical Analysis

Each polymorphism was tested for Hardy Weinberg equilibrium in the study population. An adjustment for multiple tests by Bonferroni correction was not applied in our analysis. The Bonferroni correction is commonly used by assuming that all tests are independent of each other. However in practical applications, such as our study; that is often not the case. The Bonferroni correction can be extremely conservative and leads to a high rate of false negatives, which may also contribute to publication bias [9]. Therefore Bonferroni correction in which the P-values are multiplied by the number of comparisons was not used in our analysis. Correlations between warfarin dosage and clinical and genetic factors were performed using the categoric Spearman-rank correlation with 5% two-tailed significance level. Warfarin dosage was log-transformed to normalize their distribution. Differences in the daily maintenance dose of warfarin in the different genotype groups were calculated using ANOVA with post hoc comparison using least significant difference (LSD) analysis. Multiple linear regressions were performed to model the relationship warfarin daily dose requirement with other variables measured and used to develop a novel algorithm for warfarin dosing. *P-*value <0.05 was considered statistically significant. All statistical analyses were carried out using SPSS 15 (SPSS Inc., Chicago, IL).

## Results

This is a one-centre study on inter-individual variability of warfarin dose requirements. Anticoagulant response is measured by INR. Inclusion criteria were INR readings between 1.5 and 3.0 during a minimum series of three consecutive INR measurements for a period of 3 months. The collected clinical data included gender, age, bodyweight, height and indication for anticoagulation. Clinical characteristics of the patients were listed in Table 1.

**Table 1:** Clinical characteristics of subjects

Variable	Subjects (n = 85)
Gender	
Male	47 (55.3%)
Female	38 (44.7%)
Age (yrs)	
Male	58 ± 12 (26-78)
Female	56 ± 10 (32-71)
Bodyweight (kg)	
Male	65.25 ± 10.41 (41-83)
Female	57.64 ± 14.27 (35-88)
Body height (cm)	
Male	165.74 ± 6.74 (151-180)
Female	156.10 ± 5.06 (144-167)
Body Mass Index (kg/m^2^)	
Male	23.93 ± 3.57 (15.72-36.52)
Female	23.46 ± 5.07 (15.55-32.55)
Indication for anticoagulation	
Atrial fibrillation	24 (28.91%)
Heart valve replacement	13 (15.66%)
Atrial fibrillation and heart valve replacement	6 (7.23%)
Atherothrombotic diseases	8 (9.64%)
Other	32 (38.55%)
INR	2.11 ± 0.39 (1.48-3.00)

We screened for polymorphisms in partial *CYP2C9 *locus including promoter region and regions surrounding exons 1 to 9, in addition to genetic variants in *VKORC1 *(rs9393231) and *CYP4F2 *(rs2108622). Since some reports have showed that genes other than CYP2C9, VKORC1 and CYP4F2 might also contribute to warfarin sensitivity, screening was also extended for *PROC *promoter region and intron 3, *EPHX1 *(rs4653436), *GGCX *(rs1271415) [3,8,10]. A total of 40 SNPs were evaluated in our study; 22 SNPs with Minor Allele Frequency (MAF) greater than 1% and their genotype distributions were listed in Table 2. The allele frequencies were in the Hardy Weinberg Equilibrium (HWE), except the polymorphism in *GGCX *rs12714145, which was deviated from the HWE (*P *≤ 0.05).

**Table 2:** Allele frequencies and genotype distribution of SNPs evaluated in this study

Gene	SNP	Position relative to transcription start site	Allele	n	%	MAF	Genotype	n	%	*P*-Value HWE
CYP2C9**	rs17847036	3259	G	265	98.15	0.9815	GG	130	96.30	0.8265
			A	5	1.85	0.0185	GA	5	3.70	
							AA	0	0.00	
	Novel SNP	3164	G	224	91.80	0.9180	GG	102	83.61	0.3240
	Intron 2		A	20	8.20	0.0820	GA	20	16.39	
							AA	0	0.00	
	rs9332120	3435	T	264	97.78	0.9778	TT	129	95.56	0.7917
			C	6	2.22	0.0222	TC	6	4.44	
							CC	0	0.00	
	rs2860905	3880	G	224	91.80	0.9180	GG	104	85.25	0.1556
			A	20	8.20	0.0820	GA	16	13.11	
							AA	2	1.64	
	rs28371675	3922	C	198	81.15	0.8115	CC	79	64.75	0.4291
			T	46	18.85	0.1885	CT	40	32.79	
							TT	3	2.46	
	rs28371676	3948	T	234	95.90	0.9590	TT	112	91.80	0.6369
			C	10	4.10	0.0410	TC	10	8.20	
							CC	0	0.00	
	rs28371677	4057	A	224	91.80	0.9180	AA	104	85.25	0.1556
			G	20	8.20	0.0820	AG	16	13.11	
							GG	2	1.64	
	rs9332127	9056	G	234	95.90	0.9590	GG	112	91.80	0.6369
	(*CYP2C9C-65*)		C	10	4.10	0.0410	GC	10	8.20	
							CC	0	0.00	
	rs9332129	10322	A	234	96.69	0.9630	AA	112	92.56	0.6696
			G	9	3.72	0.0370	AG	9	7.44	
							GG	0	0.00	
	rs1057910	42625	A	235	96.31	0.9631	AA	113	92.62	0.6723
	(CYP2C9*3)		C	9	3.69	0.0369	AC	9	7.38	
							CC	0	0.00	
	Novel SNP	47635	C	240	98.36	0.9836	CC	118	96.72	0.8539
	Intron 8		T	4	1.64	0.0164	CT	4	3.28	
							TT	0	0.00	
	rs2298037	47650	C	199	81.56	0.8156	CC	79	64.75	0.1957
			T	45	18.44	0.1844	CT	41	33.61	
							TT	2	1.64	
	rs9332238	50064	G	233	95.49	0.9549	GG	111	90.98	0.6020
			A	11	4.51	0.0451	GA	11	9.02	
							AA	0	0.00	
	rs1934969	50067	A	173	70.90	0.7090	AA	60	49.18	0.5595
			T	71	29.10	0.2910	AT	53	43.44	
							TT	9	7.38	
	rs1057911	50309	A	234	95.90	0.9590	AA	112	91.80	0.6369
			T	10	4.10	0.0410	AT	10	8.20	
							TT	0	0.00	
*VKORC1*	rs9923231	-1639	A	208	77.04	0.7704	AA	78	57.78	0.3027
			G	62	22.96	0.2296	AG	52	38.52	
							GG	5	3.70	
*EPHX1*	rs4653436	-2586	G	203	87.50	0.8750	GG	91	78.45	0.0633
			A	29	12.50	0.1250	GA	21	18.10	
							AA	4	3.45	
*GGCX*	rs12714145	1291	C	145	59.43	0.5943	CC	34	27.87	0.0006
			T	99	40.57	0.4057	CT	77	63.11	
							TT	11	9.02	
*PROC*	rs2069920	3643	C	140	57.38	0.5738	CC	36	29.51	0.1232
			T	104	42.62	0.4262	CT	68	55.74	
							TT	18	14.75	
*PROC*	rs1799808	-141	C	99	40.57	0.4057	CC	15	12.30	0.0563
			T	145	59.43	0.5943	CT	69	56.56	
							TT	38	31.15	
*PROC*	rs1799809	-128	A	141	57.79	0.7747	AA	72	59.02	0.3009
			G	41	16.80	0.2253	AG	46	37.70	
							GG	4	3.28	
*CYP4F2*	rs2108622	18454	C	198	81.15	0.8115	CC	82	67.21	0.3248
			T	46	18.85	0.1885	CT	34	27.87	
							TT	6	4.92	

** SNPs in *CYP2C9 *with MAF ≤ 0.01: CYP2C9*2 (rs1799853); CYP2C9*4 (rs56165452); CYP2C9*5 (rs28371686); rs17847032; rs2017319; CYP2C9*6 (rs9332131); rs9332173; CYP2C9*7; CYP2C9*8 (rs7900194); rs5031019; CYP2C9*9 (rs2256871); CYP2C9*10 (rs9332130); CYP2C9*11 (rs28371685); rs1057909; CYP2C9*12 (rs9332239); rs9332100; CYP2C9*13; rs12414460

Two reported alleles for CYP2C9, CYP2C9*2 and *3 have been identified with decreased hydroxylation activity of the enzyme, which in turn can effect the efficacy of anticoagulation [1,3]. Similar to earlier reports on other Asian populations [7,8,10,11], CYP2C9*2 carriers were not detected in our population (Table 2). We searched for additional SNPs in *CYP2C9 *locus in the promoter region of the gene, up to -700 bp upstream of the transcriptional start site, and regions surrounding exons 1 to 9. A total of 33 SNPs in *CYP2C9 *were genotyped in our study. Twelve known *CYP2C9 *allelic variants were identified in our screen (CYP2C9*2 to CYP2C9*13). CYP2C9*3 was detected with MAF greater than 1%, whereas CYP2C9*2 as well as CYP2C9*4 to CYP2C9*13 were found to be homozygous in our study population. The percentage frequencies for genotype CYP2C9*1/*1 and CYP2C9*1/*3 were 92.62% and 7.38% respectively (Table 2).

Additionally seven SNPs in *CYP2C9*, rs9332100 in promoter region, rs12414460 and rs5031019 in exon 3, rs9332173 in exon 6, rs1057909 in exon 7, rs17847032 in exon 8 and rs2017319 in exon 9, were identified as homozygous SNPs in our population (data not shown). Two novel *CYP2C9 *variants were detected in introns 1 and 8 with MAF of 8.2% and 1.64% respectively (Table 2). The two novel SNPs were detected in the position 3164 and 47635 relative to transcription start site.

Two SNPs in *VKORC1*, rs9934438 and rs9923231, have been reported to correlate with pair-wise Linkage Disequilibrium (LD) r^2 ^values of 1.0 [7], therefore only one SNP, rs9923231, was selected as candidate SNP in our study. For rs9923231 or *VKORC1 *(-1639G > A) allelic variation, 57.78% of our subjects were homozygous for the wild-type A allele, 38.52% were heterozygous and 3.7% were homozygous for the variant G allele.

Genetic variants in *EPHX1 *(rs4653436), *GGCX *(rs12714145), *PROC *(rs2069920, rs1799808, rs1799809) and *CYP4F2 *(rs2108622) were detected in our screen with MAF of 12.50%, 40.57%, 42.62%, 40.57%, 22.53% and 18.85% respectively (Table 2).

Together with non-genetic indicators such as gender, age, body weight and height, all 22 identified SNPs with MAF > 1% (Table 2) were evaluated as indicators for warfarin dose association. The non-parametric Spearman-rank correlation of warfarin dose based on the non-genetic and genetic factors is shown in Table 3. Our results indicated that among the non-genetic indicators included in the analysis, age and height were significantly correlated with warfarin dose with a *P*-value of 0.0232 and 0.0183 respectively. Bodyweight exhibited a less significant association with daily warfarin dose (*P*-value 0.0521). Among the 22 genetic indicators analyzed, only rs17847036 in *CYP2C9 *and rs9923231 in *VKORC1 *showed significant correlation with warfarin dose with a *P*-value of 0.0058 and 0.0248 respectively (Table 3).

**Table 3:** Spearman correlation of warfarin dose based on different indicators

Indicators	Spearman Correlation	*P*-value (Sig 2. tailed)	Total Samples
Gender	0.1435	0.1900	85
Age	-0.2490	0.0232	83
Bodyweight	-0.2140	0.0521	83
Body height	-0.2584	0.0183	83
*CYP2C9 *rs17847036	-0.2971	0.0058	85
*VKORC1 *rs9923231	0.2433	0.0248	85

Comparisons of age, weight, height, maintenance INR and warfarin dose were performed across the different genotypes of *CYP2C9 *and *VKORC1 *using t-test. Patients with GA genotype for *CYP2C9 *rs17847036 received significantly higher doses of warfarin (3.67 ± 0.88 mg/day; *P*-value 0.005) than patients with GG genotype (2.09 ± 0.70 mg/day). For *VKORC1*, patients carrying AA genotype for rs9923231 received lower dose albeit insignificantly (2.05 ± 0.77 mg/day; *P*-value 0.080) than patients with AG or GG genotype (2.32 ± 0.73 mg/day).

**Figure 1:**
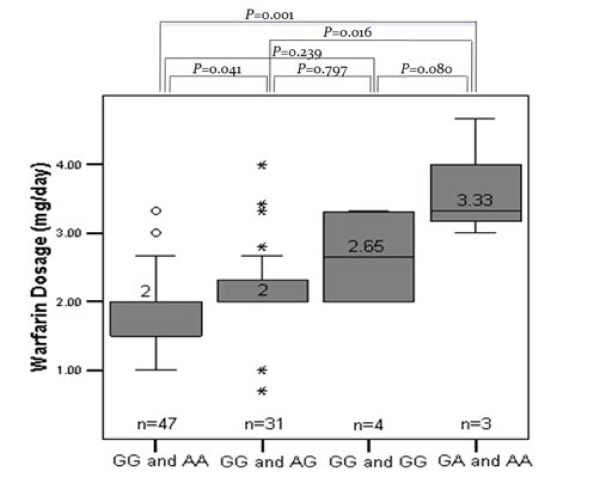
**Box plot of the distribution for warfarin dose by *CYP2C9 *rs17847036 (GG and GA) of CYP2C9 and *VKORC1 *rs9923231 (GG, AG and AA) genotypes**. Number indicates the median warfarin dose. *P*-values for the distributions in each genotype are indicated.

The variability in warfarin stable dose for patients grouped by the *VKORC1 *variants (AA, AG or GG for rs9923231) and *CYP2C9 *(AG or GG for rs17847036) is shown in Figure 1. All patients were separated into four categories according to the genotypes identified in *VKORC1 *and *CYP2C9*. The median warfarin dose for patients carrying heterozygous GA genotype for *CYP2C9 *and AA genotype for *VKORC1 *was 3.33 mg/day; higher than for patient group carrying homozygous GG genotype for *CYP2C9 *and AA genotype for *VKORC1 *with 2 mg/day (*P*-value 0.001).

**Figure 2:**
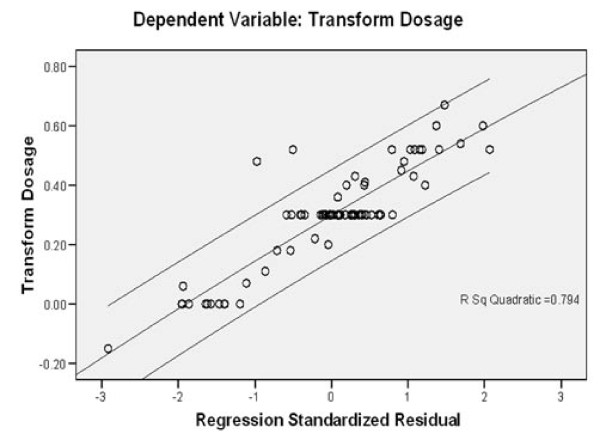
**Correlation between warfarin dose and model 4 predicted value**.

Using the non-genetic and genetic indicators, we analyzed the effect of these indicators by multiple regression model analysis as shown in Table 4. The R^2 ^value varied depending on the indicators included in the five different models used in our analysis. The model 5 showed the highest adjusted R^2 ^value of 0.154; thus, according to our analysis this model might be used to explain 15.4% of the variance in the warfarin dose. Figure 2 showed the correlation between warfarin dose and the proposed model 5. Out of 85 patients included in our analysis, our model could be used to predict the dosage of 83 patients.

**Table 4:** Multiple linear regression for estimation of warfarin daily dose requirements based on age, bodyweight, body height, *CYP2C9 *rs17847036 and *VKORC1 *rs9923231

Model	Predictors (include constant)	R^2^	Adjusted R^2^	*P*-value
1	Age, Bodyweight and body height	0.094	0.059	0.053
2	*CYP2C9 *rs17847036 and *VKORC1 *rs9923231	0.106	0.084	0.010
3	Age, Bodyweight, body height and *CYP2C9 *rs17847036	0.187	0.145	0.003
4	Age, Bodyweight, body height and *VKORC1 *rs9923231	0.107	0.060	0.069
5	Age, Bodyweight, body height, *CYP2C9 *rs17847036 and *VKORC1 *rs9923231	0.207	0.154	0.003

## Discussion

Therapy with warfarin requires frequent monitoring due to its narrow therapeutic index and inter-individual variability in the dose necessary to reach a therapeutic INR [1,12]. In this study we assessed the contribution of previously reported genetic variants to inter-individual variability in ethnic Indonesians [3,5-7,12,13]. This is the first report on the effect of genetic polymorphisms on warfarin dose requirement for this population. Based on the analysis we conclude that age, body weight, body height, and genetic variants of *CYP2C9 *and *VKORC1 *could explain for approximately 15.4% of the variability in warfarin daily dose requirement of our study population.

*CYP2C9 *metabolizes S-warfarin, the more active enantiomer of warfarin, in the liver [14,15]. In our screen for candidate SNPs in *CYP2C9*, we found that among the known *CYP2C9 *variants identified, only CYP2C9*3 was present in warfarin treated Indonesian patients. To some extent this is in agreement with published reports from other Asian populations including Chinese, Japanese, Malaysian and Korean patients [10,16,17]. Nevertheless other *CYP2C9 *genetic variants (e.g. CYP2C9*8, *11 and *13) have been reported with minor allele frequencies (MAF) as low as 1.02% in certain population [18-21]. Our analysis revealed that despite reports from other populations [3,13,22-24], CYP2C9*3 was not significantly associated with warfarin dose requirement in our study (*P*-value 0.9308, data not shown). The probable reason for this might be the low number of subjects carrying a single CYP2C9*3 allele and the absence of homozygous CYP2C9*3 individuals in our study; further analysis with a greater sample size will be required to confirm these findings.

Our study suggested a genetic variant in the exon 2 of *CYP2C9*, rs17847036, as a genetic factor that contribute to warfarin sensitivity with a *P*-value of 0.005. Together with non-genetic indicators such as patient age, body weight and body height, this genetic variant could explain about 14.5% of the inter-individual variability. To the best of our knowledge this is the first time that this polymorphism is linked with warfarin sensitivity. G/A polymorphism in rs17847036 results in a synonymous amino acid change in V76. Synonymous SNPs are not expected to alter the function of the protein; however the functional significance of these SNPs cannot be overlooked. It has been reported that synonymous mutations in coding regions may act alone or in combination with other mutations in the same transcript to influence mRNA stability and translation, thereby causing functional effects [25]. Indeed, a "silent" polymorphism in the Multi Drug Resistance (*MDR1*) gene has been attributed to alteration in the structure of substrate and inhibitor interaction sites and thus to a change in substrate specificity [26].

Recent genome-wide association studies for genetic determinants of warfarin dose have verified the involvement of CYP2C9*2 and *3 in warfarin sensitivity, and additional genetic variants of *CYP2C9 *were not significantly correlated with warfarin dose requirement [6,7]. Smaller studies however indicated that both genetic variants might not be the major predictors for warfarin dosing in African American population [27,28]. Recently, Scott et al. identified CYP2C9*8 genetic variant as the most frequent variant in African Americans, and suggested the incorporation of CYP2C9*8 into genotyping panels to improve dose prediction of *CYP2C9*-metabolized drugs, including warfarin in this particular ethnics [29]. It is therefore tempting to suggest, that *CYP2C9 *variants other than CYP2C9*2 and *3 might be associated with *CYP2C9 *enzymatic activity, and subsequently with warfarin dosing in different populations. Nevertheless additional studies in larger populations are required to confirm these findings.

Various publications have demonstrated the significant role of *VKORC1 *-1639G/A polymorphism (rs9923231) in warfarin requirements. Recent studies on *VKORC1 *suggested that SNPs in *VKORC1 *might contribute more to dose variance than SNPs in *CYP2C9 *[3,11,15,23,29]. *VKORC1 *variants have been suggested to play a role on transcriptional regulation and warfarin dose determination as haplotypes [30,31]. Indeed, Lee et al. reported that individuals carrying *VKORC1 *H1 haplotype require lower warfarin maintenance dose than individuals with *VKORC1 *H7, H8 or H9 haplotypes [32]. Moreover, these *VKORC1 *haplotypes were identified with variable frequency in Chinese, Malayan and Indian populations. Our result suggested that rs9923231 in *VKORC1 *contributed less significantly than *CYP2C9 *genetic variant rs17847036 (Table 4). From common *VKORC1 *variants only rs9923231 was analyzed in this study, this SNP was in LD with rs9934438 [13]. It is therefore conceivable that other *VKORC1 *polymorphisms might additionally contribute to dose variance in ethnic Indonesians; and this warrants further investigation. Similar to other studies, our study also suggested that patients with AA genotype in *VKORC1 *rs9923231 require lower doses of warfarin than those with AG or GG genotype [11,33].

*CYP4F2 *genetic variant rs2108622 was associated with a clinically relevant effect on warfarin requirement in Caucasian populations [34,35]. This result was confirmed by a genome wide association study, which reported that this *CYP4F2 *genetic variant might contribute to 1%-2% of the variability in warfarin dose in Caucasian populations [7]. This genetic variant was detected in our study population with a MAF of 18.85%; however this polymorphism was not significantly correlated with warfarin dose as indicated by a *P*-value of 0.9394 (data not shown). In a UK study, *CYP4F2 *rs2108622 was not associated with stable warfarin dose, but the authors reported an association between *CYP4F2 *rs2189784 and time-to-therapeutic INR, which confirmed the role of *CYP4F2 *in vitamin K metabolism [36]. A functional regulatory *CYP4F2 *haplotype has also been associated with increased susceptibility to hypertension and myocardial infarction in Chinese and Japanese population, respectively [37,38]. It is therefore tempting to suggest that additional *CYP4F2 *variants or haplotypes might affect warfarin dose requirement; and this needs further clarifications.

Similarly, unique SNPs, such as rs9332127 (*CYP2C9*C_-65_) and rs4653436 in *EPHX1*, were not strongly correlated with warfarin dose. These SNPs were reported to correlate with warfarin sensitivity in Chinese, Taiwanese and Caucasian populations [3,13,24]. The observed *P*-values for these genetic variants were 0.7304 and 0.6960 respectively (data not shown). Similar to Wang et al. [13], we were not able to detect any correlation between genetic variants in *GGCX *and *PROC *with warfarin dose requirement, despite report on other populations [3,24]. This further confirms that polymorphisms in major warfarin metabolizing genes might contribute not only to inter-individual difference within a particular population but also to inter-population variability among different populations [24].

Taken together, the present study indicated that SNPs in two major warfarin-metabolizing genes, *CYP2C9 *and *VKORC1*, contributed to the variability in warfarin dose of ethnic Indonesians. This study demonstrated the inter-individual variability in warfarin maintenance dose was due to *VKORC1 *-1639G/A polymorphism (rs9923231), *CYP2C9 *rs17847036, age, bodyweight and height in Indonesian patients. Clinical factors such as age, bodyweight and body height contributed to 5.9% of warfarin reactivity. Interestingly, the two genetic factors, *VKORC1 *-1639G/A polymorphism (rs9923231), *CYP2C9 *rs17847036, were associated with a greater contribution to warfarin sensitivity (8.4%). This clearly indicated that genetic factors play an important role in warfarin dosing in ethnic Indonesians similar to other population. Analysis of the warfarin dosing algorithm by multiple regression analysis showed that together with the non-genetic factors, the genetic predictors accounted for 15.4% of warfarin reactivity; thus this suggests that additional genetic determinants might possibly contribute to warfarin sensitivity in our study population. Genetic variants in Apolipoprotein E (*APOE*), for example, have been associated with warfarin metabolism. Polymorphisms in *APOE *gene were found to influence warfarin dose requirement differently in Caucasians, African Americans and Asians [39,40]. Recent study also identified a polymorphism in calumenin (*CALU*) (vitamin K reductase regulator) as genetic factor for warfarin requirement in African Americans [41]. Identification of additional genetic variants and further evaluation of common genetic predictors associated with warfarin sensitivity might substantially improve warfarin dose prediction for ethnic Indonesians.

## Conclusions

The study confirmed that the impacts of genetic variants on warfarin dosage requirement might vary in different population. Together with non-genetic predictors the genetic variants might be used to improve warfarin dose prediction for ethnic Indonesians.

## Abbreviations

*CYP2C9*: cytochrome P450, family 2, subfamily C, polypeptide 9; *VKORC1*: vitamin K epoxide reductase complex, subunit 1; *PROC*: Protein C; *EPHX1*: Epoxy hydrolase 1; *GGCX*: γ-glutamyl carboxylase; *ORM*: Orcosumucoid; *INR*: international normalized ratio; *CYP4F*2: cytochrome P450, family 4, subfamily F, polypeptide 2; APOE: Apolipoprotein E; SNP: Single Nucleotide Polymorphism; MAF: Minor allele frequency; LD: Linkage Disequilibrium.

## Competing interests

The authors declare that they have no competing interests.

## Authors' contributions

The study was conceived and designed by TW and IS. IS drafted the manuscript. WT carried out the molecular genetic studies and assisted in interpreting the results. AU, SR, YY and ST participated in its design and coordination. All authors have read and have approved the final manuscript.

## Pre-publication history

The pre-publication history for this paper can be accessed here:

http://www.biomedcentral.com/1471-2350/12/80/prepub

## Supplementary Material

Additional file 1 **Table S1 - List of primers used in this study**. Click here for file
