# Supplementary material for: Genetic factors associated with patient-specific warfarin dose in ethnic Indonesians
Source: BMC Med Genet. 2011 Jun 6;12:80. doi: 10.1186/1471-2350-12-80 (PMC3133537; doi:10.1186/1471-2350-12-80)
Supplement: Additional file 1 — Table S1 - List of primers used in this study. [file 1471-2350-12-80-S1.DOC]

**Additional File**

Table S1. List of primers used in this study

| **Gene** | **Region** | **Sequence (5' to 3')** | **Annealing**  **Temperature** | **Reference** |
| --- | --- | --- | --- | --- |
| *CYP2C9* | Promoter and Exon 1 | TCTCTGGTGCTGTTTCCCTTA |  | [7] |
|  |  | CAAAATACATGGTTTCATTCCACT |  |  |
| *CYP2C9* | Exon 2 | GAAGCCTGTGTGGCTGAATAA | 63.5 | ibid |
|  |  | GCTGACCATACAAACACAGTCC |  |  |
| *CYP2C9* | Exon 3 | GGAGGATGGAAAACAGAGACTT | 60 | ibid |
|  |  | TGGAAATCTGTTAGTTTGTGTGTG |  |  |
| *CYP2C9* | Exon 4 | ACTATTCTTGCCCTTTCCATCTC | 63.7 | ibid |
|  |  | TATTCACCCCAAGGCTGTCTG |  |  |
| *CYP2C9* | Exon 5 | ATCTGGTTAGAATTGATCCTCTGG | 60 | ibid |
|  |  | GTGGGCTCAGTGGTGATCTC |  |  |
| *CYP2C9* | Exon 6 | TGCCTTCCTTTTTCCCACTAA | 61.9 | ibid |
|  |  | GGAGGACACTAGCAACACCTTC |  |  |
| *CYP2C9* | Exon 7 | ACTTACCCATGCCCCTTTGT | 60 | ibid |
|  |  | ACCCGGTGATGGTAGAGGTTT |  |  |
| *CYP2C9* | Exon 8 | TTCTTCAACCTTCATGGCTTCT | 60 | ibid |
|  |  | ACAGCCTGTTCTTTCCATATCAC |  |  |
| *CYP2C9* | Exon 9 | GGTGAAGAGTAAGTATGTCCATTCA | 61.9 | ibid |
|  |  | CCCTACCCTCTTCCTCTTTGTC |  |  |
| *VKORC1* | Promoter | CAGAAGGGTAGGTGCAACAGTAA | 64 | [8] |
|  |  | CACTGCAACTTGTTTCTCTTTCC |  |  |
| *EPHX1* | 3’UTR | AAGATTGCCTCCCACACATTA | 60 | This study |
|  |  | GGGCAGTGTAGAAACAAATGC |  |  |
| *GGCX* | Intron 2 | TTGAGAACTACTGGGCTAAGGGGAC | 60 | This study |
|  |  | TGCTATATGATATGTAGAGGCGAGG |  |  |
| *PROC* | Intron 3 | GGTGTGTGTGTGACCGAAAC | 60 | This study |
|  |  | ACTGGGGAAAGAGGTGGTGG |  |  |
| *PROC* | 5’UTR | GCCACTATGGGGCTAAAATGAGA | 60 | This study |
|  |  | CATGACAGCCTGGAGTTCGAGTTAA |  |  |
| *CYP4F2* | Exon 11 | GCCGAGAAGGGAATAAAAGC | 62 | This study |
|  |  | AGGACATTGTGCTCCCAGAC |  |  |
